# Effect of COVID-19 pandemic on municipal solid waste generation: a case study in Granada city (Spain)

**DOI:** 10.1007/s10163-023-01671-2

**Published:** 2023-05-04

**Authors:** Francisco J. Peula, María Ángeles Martín-Lara, Mónica Calero

**Affiliations:** 1 NGO, Rethinking, Waste Observatory, Granada, Spain; 2 Department of Chemical Engineering, University of Granada, Granada, Spain

**Keywords:** COVID-19, Households waste, Municipal solid waste, Pandemic, Waste generation

## Abstract

The 2020 coronavirus pandemic has involved a series of political and social measures that have been adapted to the spread of the disease. Apart from the severe effects on health sector, the most effects of the pandemic have been felt in households and day-to-day life. Consequently, COVID-19 has had a significant impact on the generation of not only medical and health care waste but also of municipal solid waste production and composition. In this context, this work studied the implications of COVID-19 for municipal solid waste generation in Granada, Spain. Granada is a city where the economy is based mainly on the services sector, tourism and the University. Therefore, the impact of COVID-19 pandemic has greatly affected the city and it can be analyzed through the municipal solid waste generation. For the study of the incidence of COVID-19 in the generation of waste a period that goes from March 2019 to February 2021 has been chosen. The results show that, in the global calculation, a decrease in the generation of waste in the city is observed in this last year, reaching − 13.8%. Regarding the organic-rest fraction, the decrease in the COVID year represents − 11.7%. However, bulky waste has shown an increase in the COVID year which may be related to higher home furnishings renovation rates than in other years. Finally, glass is the waste flow that best indicates the effect of COVID in the service sector. In leisure areas, a significant reduction in the collection of glass is observed (− 45%).

**Supplementary Information:**

The online version contains supplementary material available at 10.1007/s10163-023-01671-2.

## Introduction

In early December 2019, pneumonia of unknown origin was detected in the city of Wuhan, China [1]. As a result, the Chinese health authorities were surprised by a series of pneumonia of unknown origin that had a great facility for its expansion. It did not take long to find some parallelism with the previous epidemics of severe acute respiratory syndrome coronavirus (SARS-CoV) in 2003 and Middle East respiratory syndrome (MERS) in 2012. This new epidemic caused more deaths, although with a lower lethality [2]. The causative virus, belonging to the Coronarividae family, was called severe acute respiratory syndrome coronavirus (SARS-CoV-2), and the disease, COVID-19.

At first it was thought that the epidemic outbreak could be controlled locally in China. However, on March 11, 2020, given the rapid and progressive expansion of the epidemic at the international level, the WHO decreed the status of a pandemic [3]. The virus's epicentre shifted quickly from China to Europe and, after that, to the States of America [4].

Regarding the COVID-19 epidemic in Spain, on January 31, 2020, the first case of COVID-19 was detected on the island of La Gomera (Spain), treating it as an imported case of contagion in Germany. On February 24, 2020, the first cases emerged in mainland Spain until reaching the current situation in which 3,428,354 confirmed cases have been detected with RT-PCR (reverse transcriptase polymerase chain reaction).

In 2020, the coronavirus pandemic has involved a series of political and social measures that have been adapted to the spread of the disease. On March 14, when in Spain there were around 6000 cases and 200 deaths, the Council of Ministers declared a state of alarm throughout the national territory with the aim of curbing the health emergency caused by the COVID-19 pandemic. It was initially established for a period of 15 calendar days by Royal Decree 463/2020. Then, partial lockdowns, including home quarantines and curfews, restricted/banned international and domestic travel, prohibited public gatherings, and declared emergency status to combat COVID-19.

Apart from the health sector, the most severe effects of the pandemic have been felt in households and day-to-day life [5]. For example, with the rapid rise in the number of confirmed cases, many types of COVID-19-related waste from hospitals, healthcare facilities, and individuals, including infected masks, gloves, and other protective equipment, are being generated during the pandemic. Also, COVID-19 has had a significant impact on the generation of not only medical and health care waste but also of municipal solid waste (MSW) production and composition. This is, the pandemic has transformed the waste generation dynamics [6, 7].

Although earlier studies have focused on solid waste generation at household in many countries [8-12] and other studies have analyzed the implication of COVID-19 on consumer attitudes [13, 14], medical waste generation [15, 16], plastic waste generation [17-19] and generation of other MSW, mainly from personal protective equipment or MSW management during the still-ongoing COVID-19 pandemic [7, 20-32], very little works have reported a systematic analysis with specific information on MSW generation by typology including a detailed comparative overview of MSW before and after pandemic in a particular city.

In this context, the objective of this work is to demonstrate that there is a direct relationship between municipal waste generation and the situation caused by the COVID-19 pandemic in Granada, Spain. Its novelty lies on the fact that this is the first study which investigated the different flows of household waste production in an excellent city for students and tourists. No published works have been found that have carried out a study similar to the one carried out in this work.

## Methodology

### Socio-economic aspects

The city of Granada is located in Spain, in the region of Andalusia, in the homonymous province of Granada, being its capital. With an approximate area of 8970 hectares, the municipality of Granada is integrated into a natural space coinciding with part of the La Vega are and with the peripheral reliefs that surround this Depression.

According to the official data of the municipal register published by the Statistics National Institute (INE), the population of the city of Granada is 233,648 inhabitants, which represent 25.42% of the province of Granada and 2.76% of the Andalusia population.

The city of Granada is the administrative and economic center of the province of Granada. Its economy is based mainly on the services sector, tourism and the University of Granada, which with its more than 53,000 students plays a very important socio-economic role in the city. From a demographic point of view, students constitute 20% of the population that lives in the city during scholars’ time (September–June). According to a recent study [33], the University of Granada contributes 6.12% to the Gross Domestic Product (GDP) of the province, which is equivalent to 10% for the city of Granada.

Following, the main characteristics of the different economic sectors of the municipality of Granada are analyzed. Previously, it has been identified that the service sector is the fundamental axis on which the economic development of the city revolves. Specifically, the services sector accounts for more than 80% of the total activity in Granada. Each of the economic sectors and their influence on the city of Granada are reported in Table 1.

- Farming: Currently, according to the multiterritorial information system of Andalusia, in 2019, the cultivated area was 1807 hectares, distributed in herbaceous and woody crops with an area of 1068 and 739 hectares respectively.
- Industry: Granada is not an industrial city, and neither is its province; In fact, Granada is the second province with the lowest industrial weight in Andalusia. If the Metropolitan Area of Granada had a developed industrial sector, the idiosyncrasies of the labor market and the consequences of the crisis would be very different. The industrial deficit in the capital and throughout the province, which for years has entrusted its economy to the services and construction sectors, largely determines the future of the productive activity of the territory. The latest labor market report drawn up by the Occupations Observatory of the Ministry of Employment and Social Security at the end of 2014 indicates that the economic prospects, in a territory without industry and with an enormous weight of the services sector, are not everything how flattering they should be.
- Construction: In the municipality of Granada, as in the rest of Andalusia and Spain, the sector boomed in 2008, when the construction sector represented 12.47% of the national GDP, which is equivalent to 135,659 million euros. Then, as the economic crisis progressed, its importance decreased, until it represented 5.4% of GDP in 2020.
- Services: The economic structure of the municipality of Granada is characterized by the great dependence on the services sector, an activity that is oversized compared to the regional and national set. Its growth has been much higher than the rest, and new activities have emerged such as consultancies, advertising, companies linked to the ICT sector, etc. Now, the type of services that we find in the territory is characterized by a high representation of services not intended for sale, especially administration services (education, health and defense). Next in importance are services related to tourism (hotels and restaurants) and commerce. Trade plays a very important role in the economy of the municipality. Open shopping centers and buildings dedicated exclusively to commerce and leisure are vitally important for both employment and wealth generation. The tourism sector is also one of the main pillars on which the economy and employment of the city of Granada pivot. The Alhambra and the Generalife is, without a doubt, the main attraction of the capital and the province, attracting more than two million visitors every year.

**Table 1:** Distribution percentage of the economic weight of the different productive sectors in the economy of the city of Granada.

Sector	%
Farming	1.6
Industry	5.4
Construction	6.0
Tourism (hotels and restaurants) and commerce	27.5
Administration services (education, health and defense)	27.0
Management	32.5

Own elaboration (data from 2018)

To better understand the effect of the COVID-19 pandemic on waste management, it is necessary to take into account the impact it has had on the tourism sector and university activity. For this, Table 2 shows the lockdown and restrictions on socio-economic activities timeline in Granada city during the pandemic year between March 2020 and February 2021.

**Table 2:**
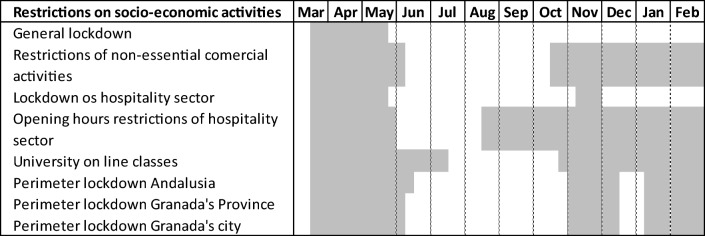
Restrictions during the analyzed pandemic period in the city of Granada

The restrictions on mobility in this pandemic year have drastically affected the number of tourists who have visited the city of Granada. According to the data provided by the Andalusia Institute of Statistics and Cartography (IECA) in 2020 there was a 70% decrease in the number of travelers compared to 2019 [34]. While in 2019 there were more than 2 million visitors, in the year 2020 only 635,000 tourists visited the city.

Considering overnight stays in hotels and tourist apartments, it is found that in 2019 there were 3,920,111 compared to 1,187,437 overnight stays in 2020, which is equivalent to a decrease of 69.7%. Transforming the number of overnight stays into equivalent inhabitants, we obtain that, in 2019, the tourists who visited the city represented an increase in the floating population of 10,740 equivalent inhabitants, compared to 3253 in 2020.

University activity is also reflected in the generation of waste in the city, the period of the academic year coinciding with the highest generation of waste. This effect is amplified with the summer vacation period, which causes a minimum of waste production in the month of August.

Mobility restrictions have led to the widespread implementation of online classes, which has caused a large number of students to have passed this course in their place of origin. Of the approximately 53,000 students, only 21% are stable residents in Granada. A large part of the non-resident students has chosen to follow the classes from their homes of origin, which has caused the city's floating population to decrease substantially compared to a normal academic year.

### Municipal waste management model

**Fig. 1:**
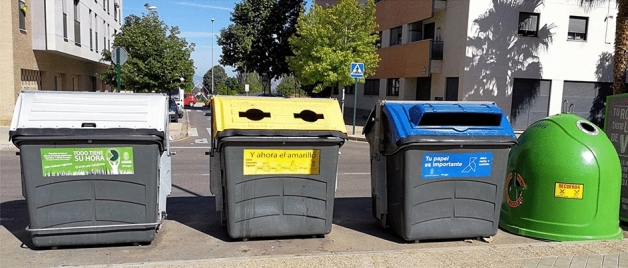
Model of 4 containers. From left to right are the containers for organic-rest waste (gray), light packaging (yellow), paper-cardboard (blue) and glass (green)

Waste management in the municipality of Granada is based on the so-called "4 container model" for the collection of the main fractions: light packaging (metal, plastic and bricks), glass containers, paper-cardboard and a container for the organic-rest fraction (organic fraction and other materials). These containers are located on public roads (Fig. 1).

Along with these four waste fractions, there are other minorities that are also collected in street containers, such as used clothing, used household oils and batteries. In addition, household goods (used furniture, appliances and other bulky waste) are collected from the public thoroughfare, which citizens deposit next to the collection containers.

Both the waste produced in homes and in those of small and medium-sized businesses are deposited in the containers on the public highway. In the case of large commercial areas, industrial estates and hospitals, they have larger containers specific to each user, which facilitates a differentiated analysis of this type of waste.

In the case of glass collection, to facilitate the work in service industry (bars, restaurants, and cafeterias), igloo-type containers are used but with a larger opening and a mechanism to facilitate the emptying of the buckets. This type of container is located in the city in leisure areas where there are plenty of bars and restaurants.

All containers in the city are equipped with geolocation systems. In turn, the collection trucks incorporate a weighing system. All this facilitates a detailed analysis of the waste generated, both by type of waste and by geographical area within the city.

### Characterization of municipal waste fractions

**Fig. 2:**
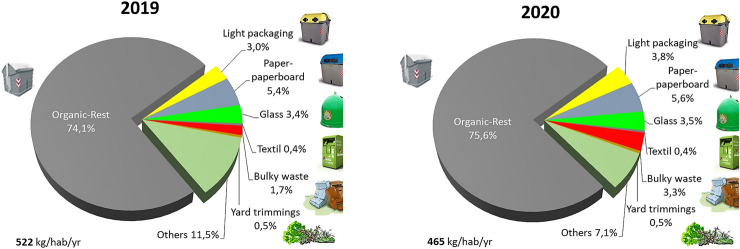
Distribution of the collection in the municipality of Granada in the years 2019 and 2020

The city of Granada generated a total of 117,882 tons of municipal waste in 2020 which were mainly treated at Ecocentral Granada, a mechanical–biological treatment facility (MBT) located in the Metropolitan Area of Granada (about 25 km from the city of Granada). This municipal waste mainly includes the fractions indicated in Fig. 2.

Therefore, MBT Ecocentral Granada receives waste fractions of organic-rest, light packaging, bulky waste and commercial/industrial waste to the recovery of recyclable materials. On the other hand, hospital waste (non-hazardous waste that can be assimilated to urban waste, such as waste from cafeterias, kitchens or offices) is disposed of in a landfill attached to the MBT. Hospital waste does not include hazardous waste of sanitary origin such as waste from operating rooms, laboratories, or nursing rooms. The paper-cardboard and glass fractions, once collected, are transported directly to recycling plants located outside the province of Granada. Table 3 shows the fractions considered and the destination of each one of them.

**Table 3:** Municipal waste fraction considered in this study and their destination

Waste fraction	Destination
Organic-rest fraction	MBT
Light packaging fraction	MBT
Bulky waste	MBT
Commercial/industrial waste	MBT
Hospital waste	Landfill
Paper-cardboard fraction	Recycling plant
Glass fraction	Recycling plant

Last 5 years, characterizations of municipal waste fractions collected by container have been carried out. During the pandemic period, these characterizations have continued to be carried out to observe possible variations in the composition of the waste.

At the entrance to the Ecocentral there is a scale where the waste is recorded according to its origin and type. In turn, the city of Granada has a control system for selective collection containers and the collection of the organic-rest fraction, based on identifying tags for the containers, which is complemented by weighing scales that are incorporated into the trucks waste collection. In this way there is a continuous record individualized for each collection container. This register facilitates the temporary monitoring of the production of the different fractions of waste, both by type of waste and by areas of the city.

On the other hand, characterizations have been carried out on the organic-rest fraction collected in the city of Granada. The samples have been extracted from the Ecocentral pit, where waste from the city of Granada is received. The extraction and homogenization procedure followed was as follows: the waste was mixed in the pit with the help of an octopus-crane; then a 2000 kg sample was taken and subjected to a quartering process with the help of a loader; a 250 kg sample was obtained for its characterization. Three samples were taken per month. The fractions considered for this characterization have been the following: plastics (including PET bottle and multilayer, PET, HDPE, PVC, film, plastics non-packaging, plastics commercial packaging and other plastics), paper and cardboard, organic matter, cellulose (including. diapers, pads and cellulose sanitary products), textile, glass, metals (including aluminum, steel and ferrorus materials) and other materials (see Table S1).

## Results and discussion

### Effects on the composition of waste

Regarding the weight distribution of the collection of the different municipal waste fractions, it can be seen in Fig. 2 how the organic-rest fraction is the predominant with respect to the other fractions, which correspond to the selective collections. Proportionally, the distribution of collections has not changed substantially between 2019 and 2020. It is worth noting the increase in the fraction of bulky waste, which has practically doubled during the year of the pandemic. All these data will be analyzed later in the following sections.

Besides, characterization of organic-rest fraction collected in the city of Granada is shown in Table 4. The composition data shown in Table 4 are the result of the annual arithmetic average of the samples characterized in the analyzed period. Results show variations depending on the materials.

**Table 4:** Composition of Granada’s organic-rest fraction in the years 2019 and 2020

Material	2019 (%)	2020 (%)	Annual variation (%)
Plastics	12.91	13.60	5.3
Metals	3.06	3.60	17.5
Paper-cardboard	16.90	16.42	− 2.9
Glass	8.16	8.21	0.6
Organic matter	32.64	32.42	− 0.7
Cellulose	6.91	5.64	− 18.4
Textiles	4.59	4.91	7.0
Others	14.84	15.21	2.5
Total	100.00	100.00	

The amount of personnel protective equipment, PPE, mainly masks and gloves, was quantified in the organic-rest fraction container throughout the summer of 2020. Spanish legislation stipulates that this waste must go in the organic-rest fraction. The amount of PPE was found to be between 0.15% and 0.28%. That is, 54 gr/inhab/month; equivalent to 7 disposable surgical masks per capita per month. In analysis of the fraction of light packaging carried out (data provided by Ecocentral Granada), it was also detected that 20% of this waste was wrongly deposited by citizens in the light packaging curbs. Regarding the content of the main components of the organic-rest fraction the average composition of the waste in the years 2019 and 2020 shows variations depending on the materials.

Some important aspects to consider are the slight increase in plastic materials (5.3%), which can be explained by the increase in packaged foods, as a measure against contamination by COVID-19. Paper-cardboard presented a slight decrease (− 2.6%), attributable to the decline in commercial activity and in domestic consumption. The glass remains practically constant (0.6%).

The component that increased the most was metals, with 17.5%. Within this group of materials, aluminum packaging stands out with a 44% rise. Aluminum in containers is closely linked to beverage cans (soft drinks and beer especially). The increase in this material is thus closely linked to the greater consumption of beverages at home, as bars were closed or had a very restrictive schedule during the pandemic.

Regarding the cellulose content, a decrease of − 18.4% is observed compared to the quantities generated in 2019. This result may be linked to the nursing homes that are important producers of this waste and that, during the pandemic, have had a specific collection to be disposed of directly in landfills.

Some of these variations suffered in the different materials could be associated with the situation caused by the pandemic and the restrictions that occurred in the city of Granada, as will be detailed in the following sections.

### Incidence of COVID in the different waste flows

For the study of the incidence of COVID-19 in the generation of waste in the city of Granada, a period that goes from March 2019 to February 2021 has been chosen. In this way, two complete annuities can be compared. The period between March 2019 and February 2020 has been called “Pre-COVID” while the pandemic year between March 2020 and February 2021 has been called “COVID”. The information shown in this section has been taken from Ecocentral Granada, a MBT located in Alhendín, Granada (Spain).

**Fig. 3:**
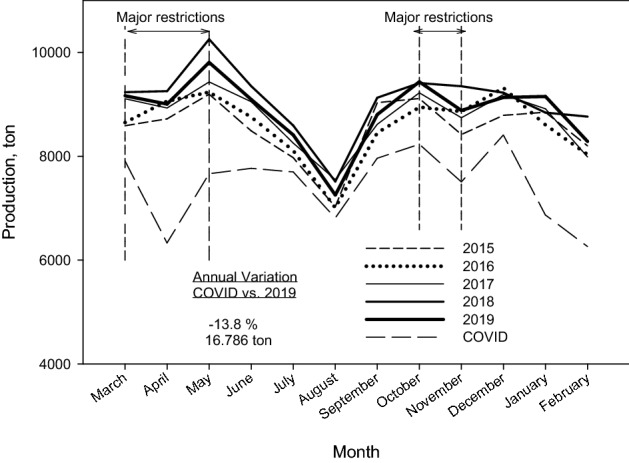
Monthly temporal changes of municipal waste production in Granada

Figure 3 shows the monthly temporal changes of municipal waste production in Granada in the last 6 years. It is observed that the behavior in the years 2015 to 2019 is very similar. However, the results show that a decrease in the generation of total waste in the city is observed in this last year, reaching − 13.8% compared to 2019. In the monthly temporal changes, it is detected that the greatest decrease has occurred during the months of confinement in the spring of 2020, when the fall was 29% in the month of April.

As shown in Fig. 3, the months with the greatest decline coincide with those with the greatest confinement and movement restrictions, the periods from mid-March to the end of May 2020, and from mid-October to the end of November 2020. In contrast, the relaxation in movement restrictions and non-essential activities led to snnoother decreases in the waste production (between 6.1% and 9.0%).

The decrease in waste production in this COVID year compared to 2019 can be related to the fall in provincial GDP. According to the study by the Bank of Spain [35] in 2020, the GDP of the province of Granada was one of the ones that decreased the most at the national level, with − 12.8%. This value is in line with the decrease in waste generation (− 13.8%).

#### Organic-rest fraction, bulky waste, paper-cardboard and light packaging

**Fig. 4:**
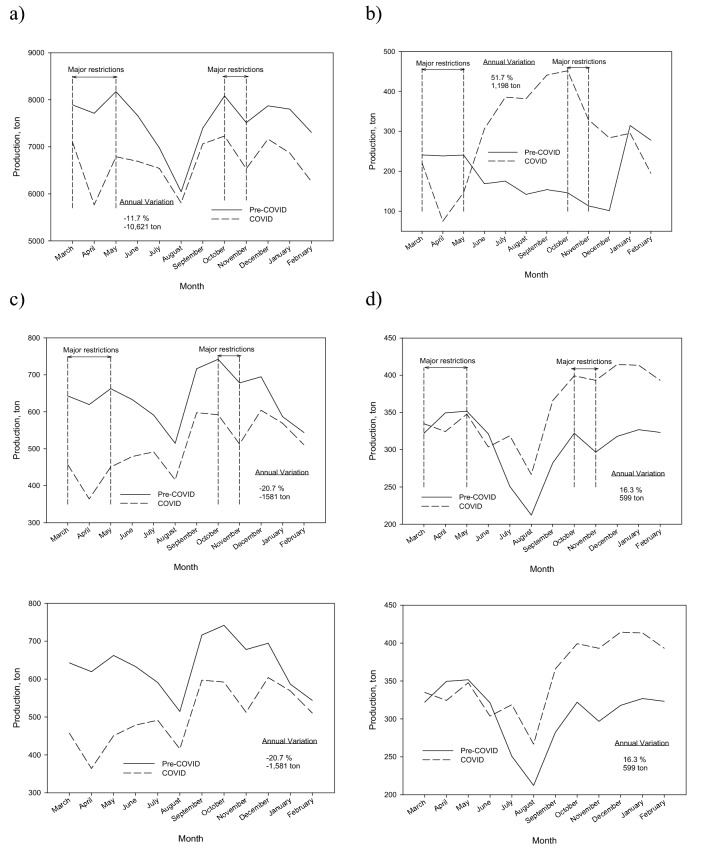
Monthly temporal changes of the production in Granada of: **a** organic-rest fraction; **b** bulky appliances; **c** paper-cardboard; **d** light packaging

The organic-rest fraction is the majority waste stream in the city of Granada as shown in Fig. 2. It constitutes about 75% of all the waste collected. Figure 4a shows the monthly temporal changes of the production of the organic-rest fraction in Granada. Although the trend is similar in both years, the decrease in the COVID year represents − 11.7%, equivalent to 10,621 tons. The monthly temporal changes reflect the periods of confinement and restrictions on movement and commercial activity. In April the fall was − 25.2% and in November − 13.1%. During the summer period there is a smaller decrease compared to the previous year, as a consequence of the lifting of restrictions.

One aspect to highlight is that the difference between the maximum and minimum production in 2019 is much greater than in the COVID year, when they are much softer. This effect can be attributed to the lower presence of students in the city in 2020. The academic year 2020–2021 began in September with face-to-face classes and went on to online classes on October 14, which caused a significant departure of non-resident students to their places of origin. This decrease in the city's floating population due to the decrease in the number of university students was also joined by the drastic drop in the number of tourists. This is reflected, for example, in the large decrease that occurred in the pre-COVID year in the month of August, which has been less drastic in the COVID year.

The flow of bulky waste (predominantly high-volume furniture and appliances) has had a temporal change in the COVID year very different from the rest of waste (Fig. 4b). After a significant decrease during the months of the first confinement (March–May), with a minimum in April of − 68.4%, there has been a sustained increase until October, when the maximum was reached, at triple the amounts collected in the previous year. In the total calculation of the year, the increase in the collection of bulky items amounts to 51.7%.

This increase in the collection of used furniture and other bulky waste has been observed in other cities in Spain. Its evolution throughout the year leads us to think of higher rates of renovation of home furnishings than in previous years.

In respect to paper-cardboard, it is a waste closely related to commercial activity. The confinement in the spring of 2020, the subsequent mobility restrictions, as well as the temporary closures of non-essential activities and the limitations in opening hours have caused that in the COVID year the collection of paper-cardboard has fallen by − 20.7%.

According to the IECA, in 2020 there was a decrease in retail sales, not related to food, of 21.5%, which would justify the decrease registered in the collection of paper-cardboard in the city of Granada.

Figure 4c shows how the annual minimum occurs in April 2020, coinciding with the closure of non-essential activities. During the summer there is a slight improvement and with the new restrictions implemented in mid-October, the collection of this material falls again.

Finally, light packaging (metal packaging, plastic packaging and bricks) is the only waste stream, along with bulky items, which has shown an increase in the last year (Fig. 4d). This increase has been detected in other cities in Spain [36-38]. In the case of Granada, the annual increase reaches 16.3%; although in the last quarter the monthly increases were around 30%.

To understand this temporal change, it is necessary to take into account the improvements that the city council has introduced in the management of the collection of this fraction. In June, the openings in the lids of the containers began to be enlarged, as well as a 30% expansion of the container park. As can be seen in Fig. 4, the effect of these measures was immediate.

It should also be noted that despite a significant decrease in the collection of the other fractions, especially in the rest fraction, during the confinement period in spring 2020 the collection of containers remained similar to that of the previous year. Therefore, in the particular case of light packaging, the observed changes cannot be attributed to the effect of the pandemic, but rather to a change in management in the collection of this waste.

#### Waste from large producers, shopping centers and hospital

The waste generated by large producers has a different collection than that used for household waste. Although Granada is a predominantly service city, it has some industrial estates whose collection is provided by private companies, with containers adapted to the needs of users.

**Fig. 5:**
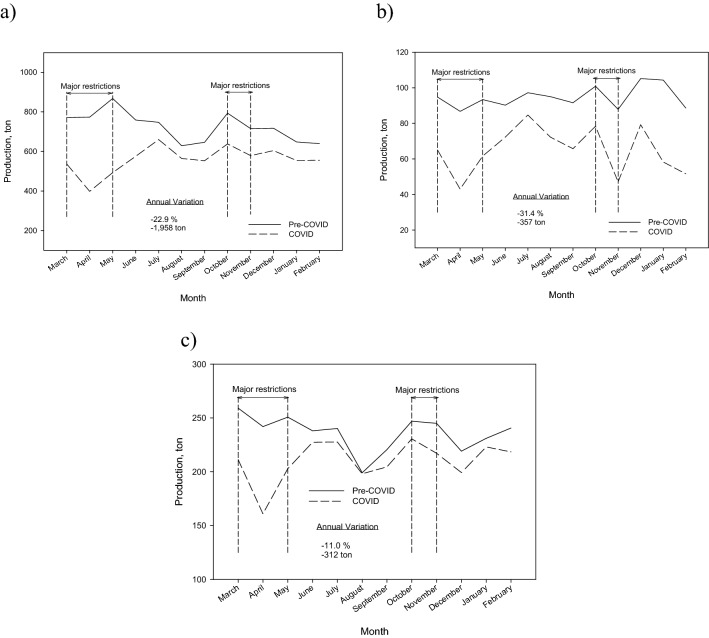
Monthly temporal changes of waste collection in Granada from: **a** large producers, **b** shopping centers and **c** hospital

Figure 5a shows the monthly temporal changes of waste collection from large producers in Granada. This waste stream has suffered a drop of − 22.9% compared to the previous year.

Also, within the large producers, two subgroups have been analyzed: shopping centers waste and type I and II hospital waste.

In the city of Granada, there are four shopping centers, whose activity has been greatly affected by the cessation of non-essential activity during the confinement of spring 2020, the perimeter closures of the municipalities and the restrictions on opening hours that have occurred over the last year.

Figure 5b shows the monthly temporal changes of waste collection from shopping centers in Granada. The generation of waste from these shopping centers has fallen on average in the COVID year by − 31.4%. According to the IECA, in 2020 sales in shopping centers in Andalusia fell by − 4.94% compared to the previous year, with an increase in food sales by 3.35% while the rest of sales fell by − 36.3%. Restrictions on service industry and leisure activities carried out inside large stores, together with restrictions on the sale of non-food products, are the main causes of the decrease in waste production.

On the other hand, Granada has an important hospital activity, with five large hospitals. Within the typology of waste generated by its activity is Type I (produced in cafeterias, kitchens and offices) and Type II (biosanitary similar to urban waste). These wastes are managed as non-hazardous waste assimilable to household waste.

Figure 5c shows the monthly temporal changes of hospital waste collection in Granada. It is necessary to clarify that hospital waste refers to non-hazardous waste that can be assimilated to urban waste, such as waste from cafeterias, kitchens or offices. The generation of hospital waste has been directly affected by the effects of the pandemic and the restrictions imposed. In the COVID year, a decrease in hospital waste has been detected by − 11%. Especially important was the drop in waste production in the spring of 2020, coinciding with the total confinement. The month of April presented a decrease of − 33.4%. During the summer, production normalized, falling again in the fall and after Christmas; coinciding with the successive waves of contagion and the consequent restrictions on mobility and hospital activity outside of COVID patients.

In the COVID year, the activity of hospital cafeterias has decreased; outpatient consultations and normal hospital activity have been reduced. According to a report from the two largest hospitals in the city [39], in 2020 surgical interventions decreased by − 27.4% compared to 2019.

#### Glass waste

**Fig. 6:**
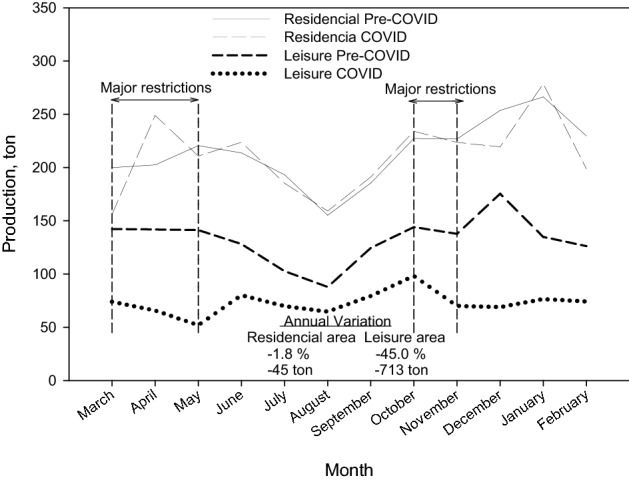
Monthly temporal changes of glass collection in Granada

Figure 6 shows the monthly temporal changes of glass collection in Granada, where it has also differentiated between mainly residential areas of the city and areas of the city with high leisure activity. Glass is the flow that best indicates the effect of COVID in the service industry (restaurant and bar sector mainly). The city of Granada, thanks to the large number of students and the tourism that visits it, has about 3500 catering establishments (coffee shops, bars and restaurants). According to the Granada Federation of Hospitality and Tourism Companies [40], this sector generates 15% of the city's GDP.

It is observed that, in residential areas, the monthly collection of glass practically coincides in the two periods analyzed (pre-COVID and COVID), which indicates that the pandemic has had little impact on the collection of glass from homes. However, if the evolution in leisure areas is observed, a significant reduction in the collection of glass is revealed (− 45%). In addition, no great peaks were found. It can be due to the lower movement of students out the city for the holidays of summer (July and August) and the lower increase of tourist in the Christmas holiday period (December). As previously indicated, the decrease in tourism and the presence of students in the city of Granada, as well as the restrictions imposed on the service sector during practically the entire COVID year, are reflected in the decrease experienced in the collection of these waste.

### An overview of waste generation during the COVID-19 pandemic

The study carried out in this work has shown that there is a direct relationship between waste generation and the situation caused by the pandemic, mainly due to the effect caused on socio-economic activity and the restrictions imposed on society.

It is difficult to compare the results obtained in this work with those found in the bibliography. First, no published works have been found that have carried out a study similar to the one carried out in this work. Second, the situation that has occurred in the city of Granada, as well as the characteristics of this city are not comparable with the studies found in other cities. Despite facing the same crisis, the situation, challenges and measures in each place could be diverse.

However, in this section, a review has been made of the works published in the last year that are oriented toward the management, generation and characterization of household waste and its relationship with the COVID-19 pandemic.

Fan et al. [21] carry out a study on waste management in response to COVID-19 in three cities with different pandemic development situations and different socio-cultural activities [Singapore, Shanghai (China) and Brno (Czech Republic)]. The authors found a variable trend in terms of the amount of municipal solid waste generated. Thus, Shanghai showed a decrease of approximately 23%, while in Singapore there was an increase of 3%. In Brno, there was a 1% increase in household waste, but a 40% decrease in industrial and commercial waste. The authors concluded that the crisis caused by the pandemic could serve as an opportunity to improve the waste management system. In addition, the authors also indicate that a wider evaluation that considers other regions of the world is necessary to establish more conclusive considerations.

Filho et al. [25] carry out an international study on the increase in household consumption and subsequent changes in the amount of waste generated since the COVID-19 pandemic. The data was collected through a survey that involved 204 consumers from 23 countries around the world and was carried out from August 2020 to November 2020. The authors concluded that the study presents evidence which shows that the restrictions caused by the pandemic caused an increase in household consumption and changes in the amount of waste generated. The changes observed in food purchasing habits had a consequence in the generation of waste. The authors indicated that according to those surveyed, the greatest increase in waste generation was observed for plastic packaging and food waste with 53% and 45%, respectively. Other types of waste such as metal and paper packaging, glass bottles and garden waste also increased their generation. However, there were no significant changes in medical or electronic waste. Respondents indicated that the change in waste generation is due to less socialization, eating at home instead of out, or having children at home instead of nurseries. Finally, the authors indicated that since the pandemic still continues, it would be useful to analyze the opinions of other stakeholders in household waste that have also experienced changes due to restrictions such as municipalities, producers or supermarkets.

Hantoko et al. [22] focused their study on changes in waste management and disposal during the COVID-19 pandemic. In addition, the authors discuss disposal technologies and alternatives to dealing with additional COVID-19 waste and propose some solutions and recommendations. The authors call attention to the increase in medical waste and infectious waste from households and review the problems in waste management and the necessary responses. To carry out the study, the authors used data from research papers, publications from organizations and governments, and media reports. The authors note that there has been a large increase in the amount of used personal protective equipment and medical waste. They also indicated an increase in the amount of food and plastic waste during the pandemic. This is in line with the increase in demand for packaging materials (PP, HDPE, PET or PS) and medical material (PP, LDPE, PVC) due to the high demand for personal protective equipment, online delivery services of food due to periods of lockdown. The study highlights the impact that all these changes have generated during the pandemic in waste management and the need to seek responses in the short, medium and long term.

Ouhsine et al. [41] carry out a study on the impact of COVID-19 on the quality and quantity of household waste, focusing on two cities in Morocco (Khenifra and Tighassaline). The authors have obtained the results through surveys of residents and compare the results during February and March 2019 and the same months in 2020. The authors concluded that the study carried out has shown that the sanitary lockdown caused by COVID-19 has had an influence on the consumption habits of citizens and, consequently, in the generation of waste. The authors indicated that the organic fraction has decreased in domestic waste, with others appearing as cleaning waste. In addition, the increase in waste generation in February and March 2019 decreased in the same period in 2020 (+ 11.41% and + 3.8%, respectively). Also, the study shows that citizens do not adequately manage personal protective equipment waste, which is mainly mixed with household waste, which represents a risk in collection.

Liang et al. [26] studied the influence of COVID-19 waste from personal protective equipment, urban solid waste and medical waste and analyze the results in different countries. With regard to medical waste, the authors indicated that there has been a significant increase, with 18–425% growth, showing data from Spain with the highest rate of increase. Also, the authors indicated that the demand for personal protective equipment will continue to increase and although the waste generated does not represent a high percentage of solid urban waste, its management should not be ignored. However, the isolation measures greatly reduced the volume of commercial waste, especially for tourist cities and part of this waste was transferred to domestic waste, due to the change in consumption and food habits produced. In this sense, lockdowns and travel restrictions have caused a drop in waste generation, especially in tourist cities. For example, the authors cited cities such as Barcelona (Spain) where the waste generation fell by 25%, Macao (China) with a 17–25% decreases or Milan (Italy) with a 28% reduction. Finally, the authors review the response to waste management and treatment systems, highlighting that many countries have adjusted their policies for waste management during COVID-19, as is the case in China. Although, the pandemic has also caused that policies related to reducing the use of plastic products or recycling waste have been stalled.

All the researches consulted coincide, as in this work, that the COVID-19 pandemic has had an effect on the quantity and composition of household waste. In general, mobility restrictions, lockdown periods and limitations in sectors such as the hospitality have been reflected in the generation of waste. However, studies showed different trends regarding municipal solid waste. Furthermore, these trends largely depend on the socio-economic characteristics of the cities, regions or countries in which the study has been carried out.

Recently, ACR+ has published a report on the impact of the COVID-19 pandemic on municipal waste management systems [42]. The study has been carried out through surveys conducted in 10 different countries in terms of type, size or tourist activity. The study has focused on the period between February and June 2020. Regarding the impact of the pandemic on the generation of municipal waste, the report indicates that the impact differs from one site to another, although the most common trend is a decrease in the amounts of waste generated, which coincides with what found in this work. This decrease is attributed, according to the report, to the decrease in commercial waste and, in some cases, to the effect of the significant drop in tourism. This coincides with what was found in this work since Granada is a city where tourism and the University represent an important part of its economy, which has had a significant impact on the generation of waste.

The report also includes results found in other studies. For example, the report shows data on amounts of waste collected in the Region of Catalonia (Spain) and the city of Milan (Italy). In both territories, the amounts of waste decreased, with a significant decrease in paper-cardboard and glass in Catalonia and commercial food waste in Milan. This decrease can be due to the closure of commercial activities and the service sector. They also indicate that other studies have reported an increase in some types of waste such as bulky waste, construction and demolition or garden waste, which could be related to small renovation works in homes carried out by the citizens during the lockdown periods. Similar results have been obtained in this work since, as indicated in Sect. “Incidence of covid in the different waste flows”, a significant increase in bulky waste was observed (Fig. 5).

The study carried out has shown that the COVID-19 crisis also had its own effect on the circular economy, with disruptions in recycling activities and changes in consumer and firm behavior. Some recycling activities, such as manual sorting have temporarily come to a halt, border closures disrupted recycling supply chains and increased online shopping and take-away orders, as well as the regular use of personal protective equipment (PPE) have led to an increased consumption and waste generation from single-use plastic items. The COVID-19 crisis can be an opportunity for cities to rethink urban policies toward more sustainable production and consumption patterns.

## Conclusions

In this study, the scenario of the current municipal waste generation in Granada (Spain) throughout the pandemic period has been reported and compared with pre-covid data. Regards the global composition of waste, there was an important increase in metals (17.5%) and a slight rise in percentage of plastic materials (5.3%), while paper-cardboard shows a slight decrease (− 2.6%) and the cellulose content a significant decrease (− 18.4%). These differences found in the different materials were connected with the situation caused by the pandemic and the restrictions that occurred in the city of Granada. In the context of current pandemic situation in Granada, an important decrease of − 11.7% was observed in organic-rest fraction, as a consequence of the lifting of restrictions. High-volume furniture and appliances showed a significant decrease during the first confinement with a minimum in April of − 68.4% and then, until October, a constant increase was observed. The generation of waste shopping centers and hospitals fallen and an important decrease of − 20.7% in paper-cardboard was observed. Also, the pandemic has had little impact on the collection of glass from homes. However, the decrease in tourism and the presence of students in the city of Granada, as well as the restrictions imposed on the service sector during practically the entire COVID year, reflected a decrease in the global collection of glass waste.

The study carried out has shown that there is a direct relationship between the generation of waste and the situation caused by the pandemic. This relationship is mainly due to the restrictions imposed that have caused a decrease in socio-economic activity. This study can be used to establish comparisons with other cities in the world with characteristics similar to the one analyzed in this work, which will allow establishing more conclusive conclusions.

## Supplementary Information

Below is the link to the electronic supplementary material.

Supplementary file1 (DOCX 19 KB)

## Data Availability

All data and materials comply with field standards.
