# Supplementary material for: Effect of COVID-19 pandemic on municipal solid waste generation: a case study in Granada city (Spain)
Source: J Mater Cycles Waste Manag. 2023 May 4:1–13. Online ahead of print. doi: 10.1007/s10163-023-01671-2 (PMC10156574; doi:10.1007/s10163-023-01671-2)
Supplement: Supplementary file 1 — Supplementary file1 (DOCX 19 KB) [file 10163_2023_1671_MOESM1_ESM.docx]

**SUPPLEMENTARY MATERIAL**

**EFFECT OF COVID-19 PANDEMIC ON MUNICIPAL SOLID WASTE GENERATION: A CASE STUDY IN GRANADA CITY (SPAIN)**

**Authors**: Francisco J. Peula, María Ángeles Martín-Lara*, Mónica Calero

Table S1. Complete characterization of Granada's organic-rest fraction in the years 2019 and 2020

| **MATERIAL** | **2019** | **2020** |
| --- | --- | --- |
| PET Bottel | 1.85% | 2.17% |
| PET Multilayer | 1.00% | 0.98% |
| HDPE | 0.90% | 1.12% |
| PVC | 0.05% | 0.10% |
| Film | 5.31% | 5.24% |
| Plastic personal protective equipment | 0.15% | 0.28% |
| Other plastics | 1.64% | 1.72% |
| Plastics non-packaging | 1.78% | 1.77% |
| Plastics commercial packaging | 0.24% | 0.22% |
| **Total Plastics** | **12.91%** | **13.60%** |
|  |  |  |
| Paper and cardboard | 16.90% | 16.42% |
| Organic matter | 32.64% | 32.42% |
| Cellulose | 6.91% | 5.64% |
| Textile | 4.59% | 4.91% |
| Glass | 8.16% | 8.21% |
|  |  |  |
| Aluminiun packaging | 0.74% | 1.06% |
| Aluminium non-packaging | 0.03% | 0.03% |
| Ferrorus non-packaging | 0.68% | 0.98% |
| Steel packaging | 1.61% | 1.53% |
| **Total metals** | **3.06%** | **3.60%** |
|  |  |  |
| Wooden non-packaging | 1.34% | 1.66% |
| Commercial wooden packaging | 0.02% | 0.13% |
| Bricks | 1.30% | 1.21% |
| Wooden packaging | 0.21% | 0.07% |
| E-Waste | 0.55% | 0.79% |
| Hazardous waste | 0.12% | 0.19% |
| Inert waste | 1.73% | 3.24% |
| Fines | 9.54% | 8.06% |
| **Total Others** | **14.80%** | **15.36%** |
| **TOTAL** | **100.00%** | **100.00%** |
